# Perception of climate change in patients with chronic lung disease

**DOI:** 10.1371/journal.pone.0186632

**Published:** 2017-10-18

**Authors:** Jeremias Götschke, Pontus Mertsch, Michael Bischof, Nikolaus Kneidinger, Sandhya Matthes, Ellen D. Renner, Konrad Schultz, Claudia Traidl-Hoffmann, Hans-Werner Duchna, Jürgen Behr, Jürgen Schmude, Rudolf M. Huber, Katrin Milger

**Affiliations:** 1 Department of Internal Medicine V, Comprehensive Pneumology Center (CPC-M), Member of the German Center for Lung Research (DZL), University of Munich, Munich, Germany; 2 Chair of Economic Geography and Tourism Research, Department of Geography, University of Munich, Munich, Germany; 3 Christine Kühne - Center for Allergy Research and Education, CK-CARE, Davos, Switzerland; 4 Chair and Institute of Environmental Medicine, UNIKA-T, Technical University Munich and Helmholtz Zentrum München - German Research Center for Environmental Health, Augsburg, Germany; 5 Hochgebirgsklinik Davos, Davos, Switzerland; 6 Klinik Bad Reichenhall der Deutschen Rentenversicherung Bayern Süd, Bad Reichenhall, Germany; 7 Asklepios Fachkliniken München Gauting, Gauting, Germany; Telethon Institute for Child Health Research, AUSTRALIA

## Abstract

**Background:**

Climate change affects human health. The respective consequences are predicted to increase in the future. Patients with chronic lung disease are particularly vulnerable to the involved environmental alterations. However, their subjective perception and reactions to these alterations remain unknown.

**Methods:**

In this pilot study, we surveyed 172 adult patients who underwent pulmonary rehabilitation and 832 adult tourists without lung disease in the alpine region about their perception of being affected by climate change and their potential reaction to specific consequences. The patients’ survey also contained the COPD Assessment Test (CAT) to rate the severity of symptoms.

**Results:**

Most of the patients stated asthma (73.8%), COPD (9.3%) or both (11.0%) as underlying disease while 5.8% suffered from other chronic lung diseases. Patients and tourists feel equally affected by current climate change in general, while allergic subjects in both groups feel significantly more affected (p = 0.04). The severity of symptoms assessed by CAT correlates with the degree of feeling affected (p<0.01). The main disturbing consequences for patients are decreased air quality, increasing numbers of ticks and mosquitos and a rising risk for allergy and extreme weather events such as thunderstroms, while tourists are less disturbed by these factors. Increasing number of heat-days is of little concern to both groups.

**Conclusion:**

Overall patients are more sensitive to health-related consequences of climate change. Yet, the hazard of heat-days seems underestimated and awareness should be raised.

## Introduction

Ongoing climate changes already affect human health and the consequences are predicted to increase in the next decades [1,2]. Breathing around 10.000 litres of the surrounding air per day, the lung is permanently exposed to the environment and thus among the first organs affected by environmental changes.

The prevalence of respiratory diseases, especially asthma and COPD is high and increasing worldwide [3]. Patients with such respiratory diseases are particularly vulnerable regarding environmental alterations associated with climate changes [4]. For example, heat-wave-related mortality has been found to be higher among those with chronic respiratory disease, even when hospitalised [5]. Heat stress leads to an excess mortality risk of up to 43% in patients with chronic lung disease [6].

One underlying mechanism may be the hot and humid air found on heat days known to trigger asthma attacks [7]. Further, warming temperatures also affect air quality. Air pollutants like ozone, nitrogen dioxide or sulphur dioxide increase in warming temperatures with elevated risk of asthma exacerbations [8]. Likewise patients with COPD suffer from exacerbations more frequently in case of heat-days and increased air pollution [9,10]. Additionally, air pollution does not only promote exacerbation of existing respiratory disease, it is also a causative factor [8,11].

Besides, climate changes raise the risk of a variety of infectious diseases, including respiratory infections and those transmitted by vectors such as mosquitos and ticks [12].

Moreover, extreme weather events like thunderstorms are related to outbreaks of asthma exacerbations probably by increasing inhalable allergen load in the air [4].

Finally, with global warming the number and size of plants is increasing, as well as the amount of pollen and allergenic proteins produced by each plant resulting in an overall elevated risk of pollen allergies [13]. Global warming may also lead to prolongation of the pollen season [14–16] and occurrence of new allergens [17] in temperate zones, rendering asthma therapy and control more difficult in affected patients. Importantly, pollen is also increasingly found in regions that used to be nearly free of pollen, like higher altitudes in the Alps [18]. The alpine region has been a classical location for pulmonary rehabilitation mainly because of clean air. A recent meta-analysis found that high-altitude alpine therapy is beneficial in asthmatics [19]. Availability of this therapeutic principle may be at risk when alpine climate changes. In addition to clean air, the landscape is the main attraction of the Alps and climate warming-induced changes hereof are particularly obvious with melting glaciers, less snow and different vegetation. Thus, tourists are a second population that may be affected by the changes, even in absence of chronic disease.

Recently, there have been an increasing number of studies investigating the influence of climate change on clinical outcomes of patients and on the well-being of the general population. Parameters studied included lung function, symptoms, hospital admissions and mortality. However, the awareness and individual reactions of patients with respiratory disease to the ongoing climate change in the alpine region has not been studied so far. Yet, such patient-centred outcomes are important in order to develop strategies for information and intervention in this vulnerable population.

## Methods

From November 2015 to August 2016, we surveyed 172 adult patients who underwent pulmonary rehabilitation in the alpine region, thereof 58 in Davos, Switzerland, and 114 in Bad Reichenhall, Germany. On two other occasions in May and August 2015, further 832 healthy tourists aged ≥ 18years were surveyed in the alpine region, thereof 614 in the city of Meran, Italy, and 218 in Garmisch-Partenkirchen, Germany. Recruitment of patients took place “on the streets” near touristic attractions like cable car station or city centre, recruitment of patients took place within the rehabilitation clinics. The surveys for tourists and patients were equally structured except for an additional part in the patients' survey containing the CAT score. Patients were asked to retrospectively fill out the CAT score how they felt at home immediately before the rehabilitation and now at the end of the rehabilitation. Surveys were in German language and can be assessed as German and English version in the online supplement (S1 and S2 Files). All surveys were filled out anonymously. The study was approved by the institutional review board (IRB) of the University of Munich (Nr.129/16).

A significance level of 5% was used for all statistical tests. All analyses were performed using the statistical software SPSS (Version 24 IBM). Normality testing by visual method and Shapiro-Wilk Test to test found that most of the data were not normally distributed. Therefore Kruskal-Wallis-Test was used to test if two independent groups differed with respect to a metric variable. For correlation analysis Spearman’s rho coefficient was calculated. Fisher’s Exact Test was applied for contingency tables. No adjustment of p-values for multiple testing was performed, as this was considered a pilot-study.

The tourist survey was also part of a larger study on the consequences of climate change in the alpine region on tourism. In the present study, the data from tourists was only used as control to rate the patients’ perception. Therefore only tourists who had never been diagnosed with lung disease were included in this analysis. A detailed analysis of the data from all tourists will be the subject of a different report focussing on tourism. For all analyses using the CAT score we included only patients with asthma and COPD as the questionnaire has not been studied in other diseases.

## Results

A total of 172 patients and 832 tourists were included in the study. The mean age was 51.7 years in both groups and there were slightly more men included in both groups. Most of the patients stated bronchial asthma (73.8%) or COPD (9.3%) or both (11.0%) as underlying disease, while 5.8% were affected by non-obstructive lung diseases like different forms of interstitial lung disease and pulmonary hypertension (Table 1). Mean age of patients with asthma and patients with asthma + COPD was less than the age of patients with COPD or other lung diseases (50.11 and 52.66 years versus 58.87 and 59.50 years respectively, Table 1). Presence of allergy was indicated by the majority of patients (73.3%), while less than one third of tourists (30.8%) stated to be allergic. If specified, allergy to pollen was the most frequent type of allergy in both groups.

**Table 1:** Characteristics of patients and tourists.

	Patients	Tourists
N	172	832
Male %	51.2	53.9
Female %	48.8	46.1
Age -years (sd)	51.72 (10.8)	52.45 (14.7)
Atopic rhinitis %	76 (44.4%)*	181 (21.9%)*
**Chronic lung disease**	172	0
Asthma	127 (73.8%)	-
COPD	16 (9.3%)	-
Asthma+COPD	19 (11.0%)	-
Others^+^	10 (5.8%)	-
**Age acc. to lung disease- yr (sd)**		
Asthma	50.11 (10.97)	-
COPD	58.87 (6.78)	-
Asthma+COPD	52.65 (9.16)	-
Others^+^	59.50 (8.96)	-
**Allergy**	126 (73.3%)*	255 (30.8%)*
house dust mite	58	18
Pollen	88	146
Animal	49	20
Food	36	18
Others/Not specified	12	27
**Smoking**		
active	16 (9,3%)	88 (10.7%)
Ex	61 (36%)	738 (89.3%)^#^
Never	95 (55%)	
Education Level (1–8)	3.49*	4.79*

^+^ other chronic lung diseases included various forms of interstitial lung disease and pulmonary hypertension

* significantly different between patients and tourists (p<0.05 by Fisher’s Exact Test)

^#^ ex + never smoker combined

Education level: 1- no qualification, 2- primary school, 3- Secondary school (GCSE level), 4- advanced technical college entrance qualification, 5- diploma from German secondary school qualifying for university admission, 6- college diploma, 7- university diploma, 8- other e.g. doctoral degree.

**Fig 1:**
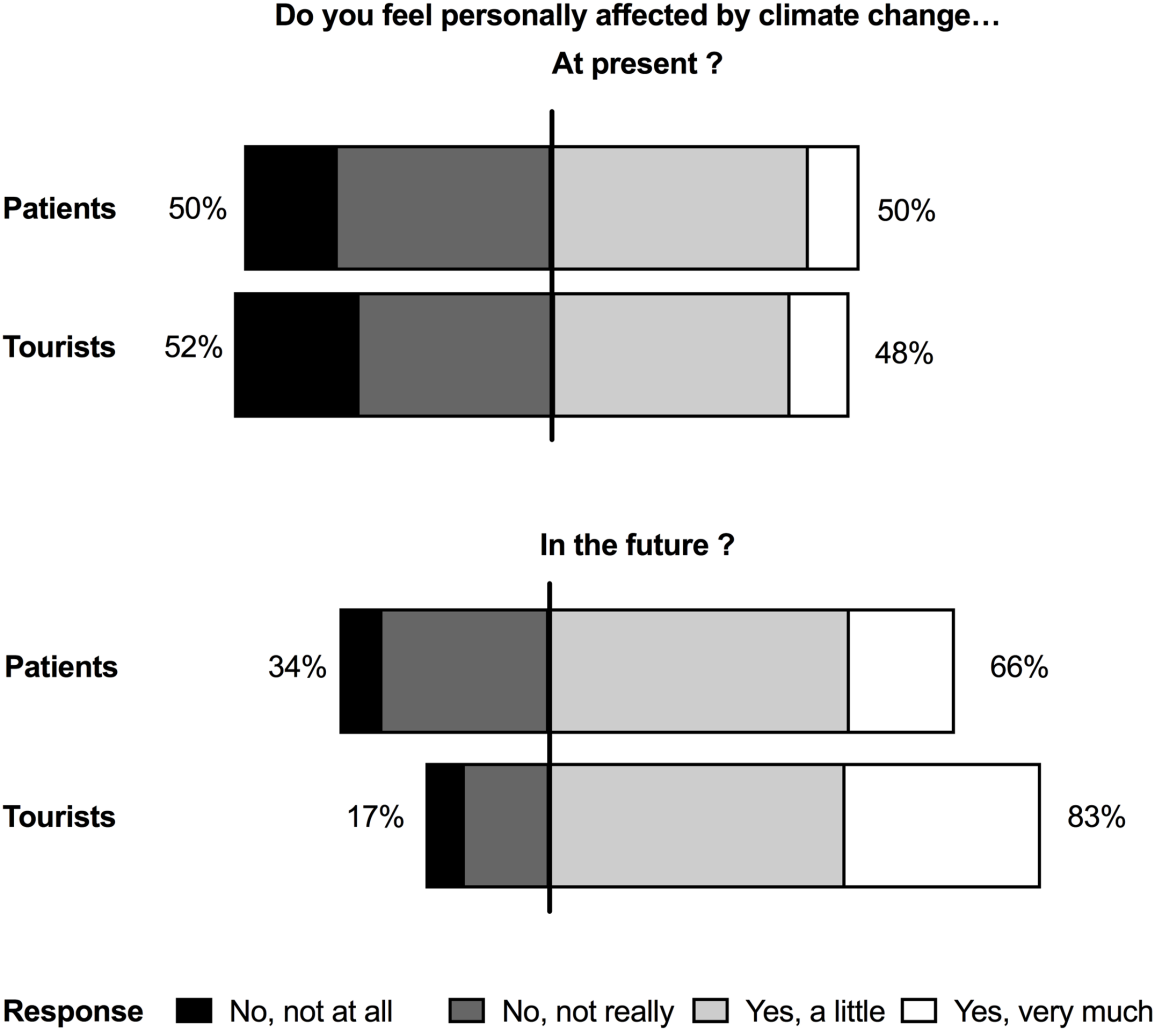
General perception of being affected by climate change in patients with lung disease and tourists without lung disease at present and in the future. Both groups anticipated to be significantly more affected in the future than at present (Fisher’s Exact test, p<0.001). In the future tourists feel more affected than patients (Fisher’s Exact test, p<0.001).

Patients and tourists were asked to rate how much they feel generally affected by the climate change at present and in the future (1- not affected at all, 2- rather not affected, 3- rather affected, 4- very affected). At present, patients with lung disease and tourists without lung disease feel equally affected (2.38+/- 0.91 vs. 2.44 +/-0.84, Fig 1). Both groups anticipated to become significantly more affected in the future than at present (p<0.001, Fig 1). Tourists, however, expect to feel more affected in the future than patients (3.13 +/- 0.82 vs 2.77 +/- 0.81; p<0.001).

Moreover, we investigated the correlation of socio-demographic and disease characteristics of patients with the perception of being affected by climate change in general. Here we found that patients with allergies feel more affected by climate change at present and in the future than those without allergies (Table 2).

**Table 2:** Correlation of parameters with patients’ perception of being affected by climate change at present and in the future.

	At present	In the future
	p-value	p-value
	(correlation coefficient)	(correlation coefficient)
**Allergy**	0.042^K^	0.017^K^
**Allergic rhinitis**	0.088^K^	0.384^K^
**COPD Assessment Test**		
**Current (end of rehabilitation)**	0.005^S^ (0.207)	0.057^S^ (0.139)
Cough	0.334 (0.071)	0.673 (0.031)
Phlegm	0.607 (0.038)	0.645 (0.034)
Chest tightness	0.026 (0.163)	0.184 (0.097)
Dyspnea	0.068 (0.134)	0.205 (0.093)
Loss of confidence	0.004 (0.212)	0.026 (0.163)
Sleep quality	0.016 (0.176)	0.151 (0.106)
Energy level	0.132 (0.111)	0.377 (0.065)
**At home (before rehabilitation)**	<0.001^S^ (0.258)	<0.001^S^ (0.245)
Cough	0.178 (0.098)	0.085 (0.125)
Phlegm	0.080 (0.273)	0.019 (0.171)
Chest tightness	0.001 (0.245)	<0.001 (0.263)
Dyspnea	0.050 (0.143)	0.173 (0.099)
Loss of confidence	0.001 (0.230)	0.008 (0.191)
Sleep quality	0.001 (0.243)	0.013 (0.180)
Energy level	0.002 (0.227)	0.007 (0.196)
**Age**	0.415^S^ (-0.060)	0.741^S^ (-0.024)
**Education**	0.364^S^ (0.066)	0.092^S^ (0.122)
**Sex**	0.935^K^	0.569^K^

p<0.05 are considered significant and marked in bold. Correlation coefficient in brackets if applicable. Statistic tests used:

^K^ Kruskal-Wallis-Test for categorical variables,

^S^ Spearman rank correlation for numerical variables.

**Fig 2:**
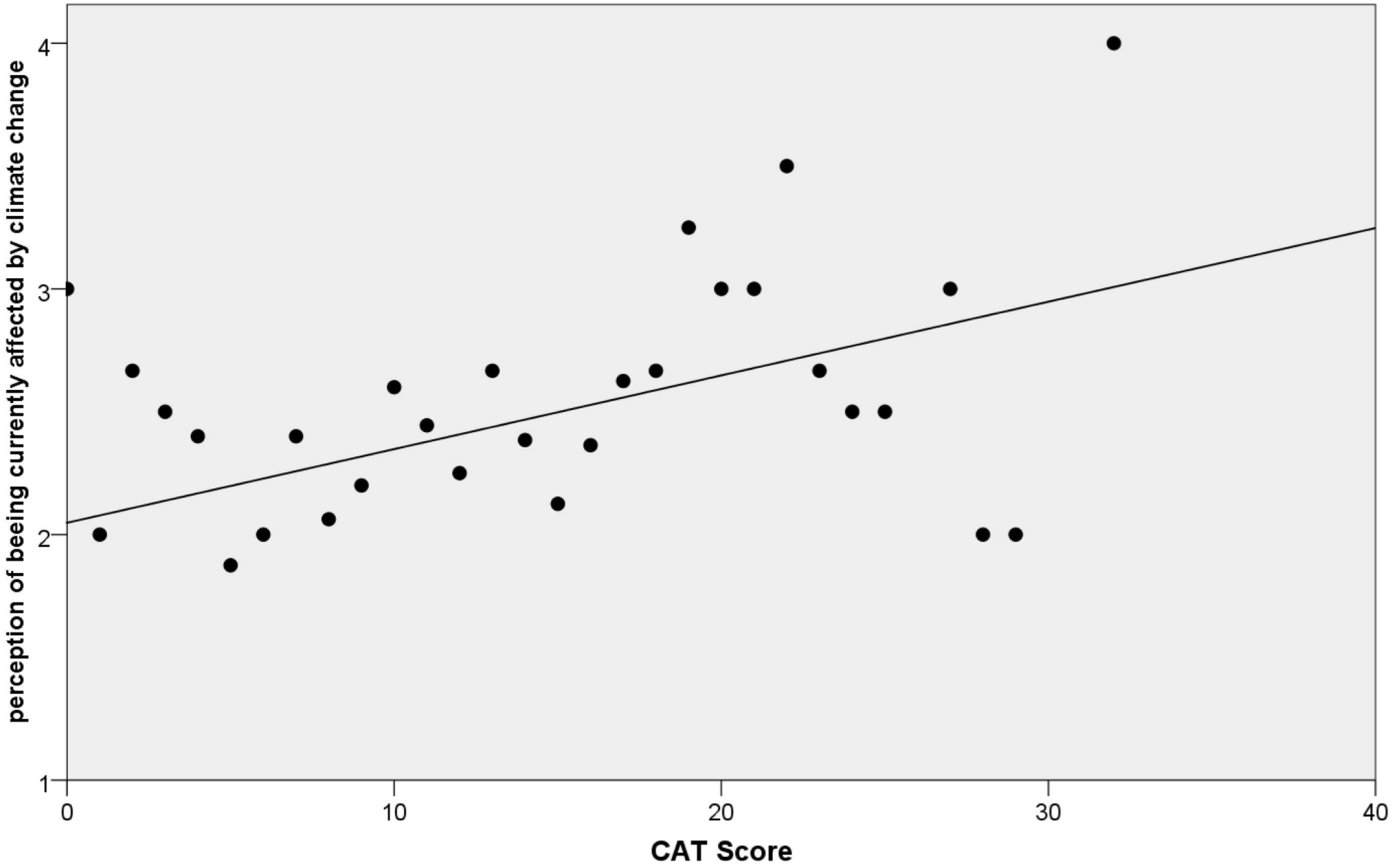
Correlation between CAT score and perception of being currently affected by climate change (p<0.01, r = 0.222 by Spearman's Rank-Order Correlation).

When looking at patients with allergic rhinitis there was a trend (p = 0.088). Furthermore, there was a significant correlation between the CAT score at home as well as at the end of rehabilitation and the perception of being currently affected by climate change (Table 2, Fig 2). Looking at specific items of the CAT score, we found significant correlations for the items chest tightness, loss of confidence and sleep quality during rehabilitation. In addition to these items, at home there was also a correlation to the energy level (Table 2). When comparing the CAT score at home with the one at the end of rehabilitation, we found a significant decrease in score and the amount of decrease correlated with higher initial CAT scores (p<0.001, r = 0.511) while there was no correlation with socio-demographic factors, current smoking status or the type of lung disease.

In patients, we also found that those with allergies and allergic rhinitis feel more affected by climate change at present (p = 0.044 and p = 0.036), while for the future there was a trend (p = 0.097 and p = 0.089).

**Fig 3:**
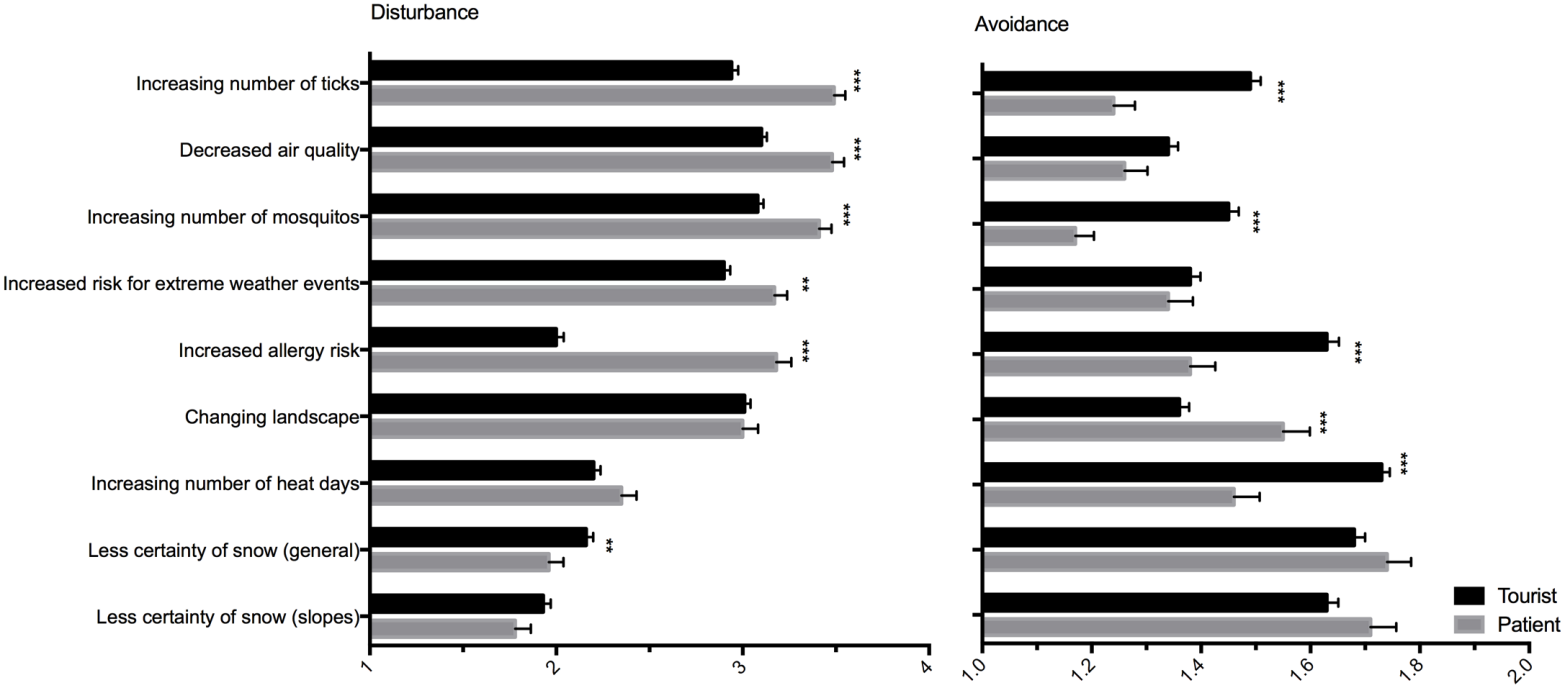
Disturbance and avoidance of specific consequences of climate change in patients and tourists. Left: Degree of disturbance. Rating: 1- not disturbing, 2- rather disturbing, 3- disturbing, 4 absolutely disturbing. Right: Avoidance. Rating: 1- not avoid, 2- avoid. The item extreme weather events include thunderstorms, gale storms and heavy rain, but not heat days. * p<0.05, ** p<0.01, *** p<0.001 significant differences between patients and tourists by Kruskal-Wallis-Test.

Next, we looked at specific consequences of the climate change that might be important for patients and tourists in terms of health and attractiveness of the alpine destination. Subjects were asked to rate the degree of disturbance (1- not disturbing, 2- rather disturbing, 3- disturbing, 4- absolutely disturbing) for each factor. It was further inquired whether each disturbing factor (rating ≥2) would lead to avoidance of the alpine region as a destination. Changes of weather and natural scenery—such as increasing number of heat days, loss of snow certainty and changing landscape, are equally disturbing to patients and tourists (Fig 3). Yet patients feel significantly more disturbed by increased risk of extreme weather events such as thunderstorms, increased allergy risk, increasing number of mosquitos and decreased air quality. The latter factors are also those with the highest absolute rate of disturbance in the patients (Fig 3).

Concerning the reaction to these disturbing factors in terms of avoidance, a significantly higher proportion of patients would avoid increased number of ticks and mosquitos and decreased air quality but also heat days, whereas significantly more tourists would avoid regions with changing landscape and less certainty of snow (Fig 3).

**Fig 4:**
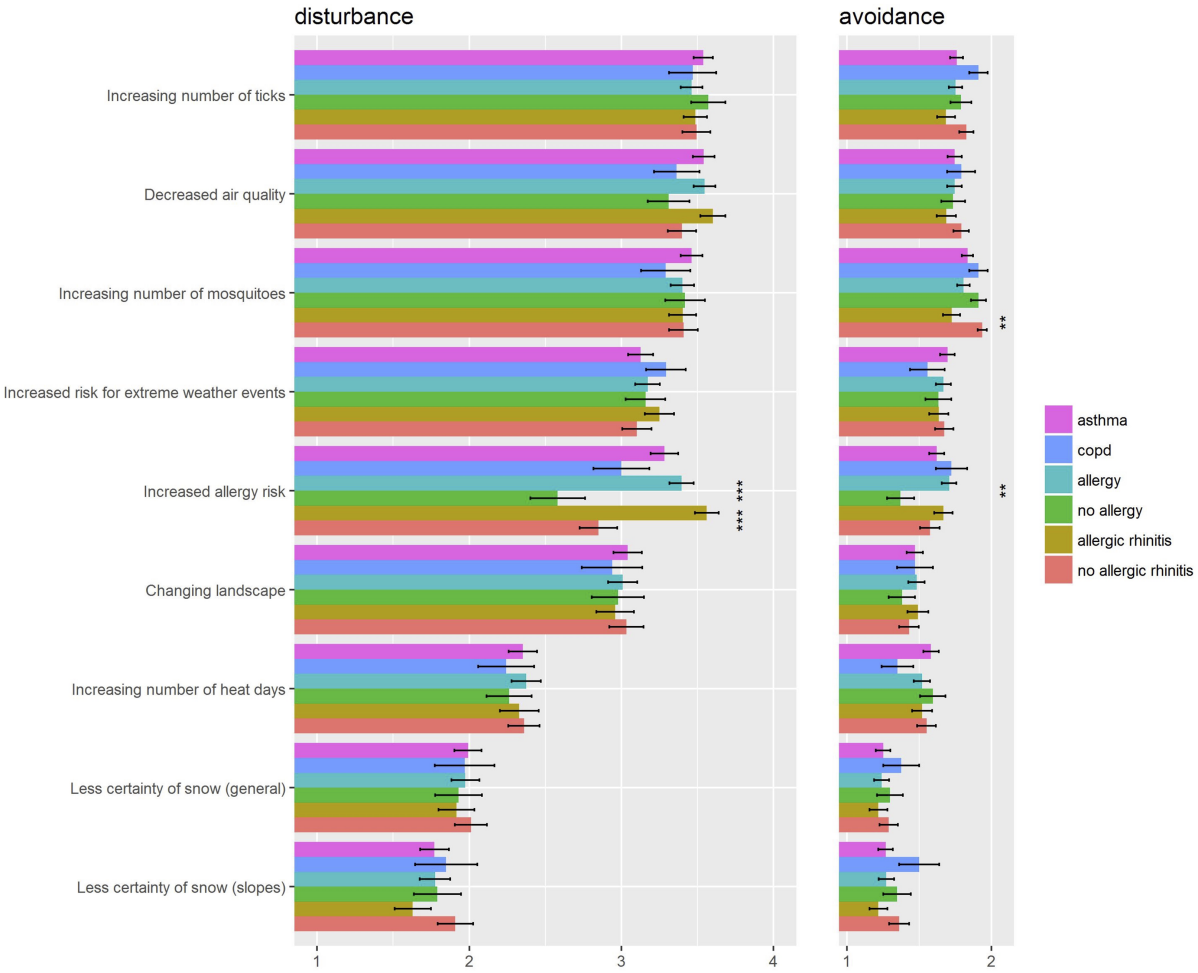
Disturbance and avoidance of specific consequences of climate change in patients classified according to underlying lung disease, allergy and allergic rhinitis. Left: Degree of disturbance. Rating: 1- not disturbing, 2- rather disturbing, 3- disturbing, 4 absolutely disturbing. Right: Avoidance. Rating: 1- not avoid, 2- avoid. The item extreme weather events include thunderstorms, gale storms and heavy rain, but not heat days. * p<0.05, ** p<0.01, *** p<0.001 significant differences between categories (Asthma vs COPD^#^, Allergie vs No Allergy, Allergic rhinitis vs No allergic rhinitis) by Kruskal-Wallis-Test. ^#^ COPD group includes patients who stated COPD or COPD+Asthma as diagnoses.

For the patients, we further analysed whether degree of disturbance and reaction to specific consequences of climate change differed according to the type of lung disease (asthma vs. COPD/ COPD+asthma) or the presence of allergies and allergic rhinitis (Fig 4). Patients with allergies or allergic rhinitis feel significantly more disturbed by an increased allergy risk than those without this kind of disease. Those with allergies would also avoid such risk locations to a significantly higher percentage (Fig 4). Moreover, significantly more patients with allergic rhinitis would avoid increasing numbers of mosquitos. For all other factors the patterns were similar among all groups (Fig 4). Finally, patients who are more symptomatic as assessed by CAT score, feel more disturbed by extreme weather events such as thunderstorms (p = 0.06, r = 0.141).

As presence of allergies was an important factor, we also compared patients with allergies to tourists with allergies. Here the present concern of patients and tourists with allergies did not differ (S1 Fig). However, regarding the future tourists feel more affected by climate change than patients. When comparing to the whole patient population (compare S1 Fig and Fig 1), allergic patients and tourists display more general concern at present as well as in the future.

Further we also looked at specific items of disturbance and avoidance in allergic subjects. Allergic patients feel significantly more disturbed by increased allergy risk, decreased air quality, increased risk for extreme weather events and increasing numbers of mosquitos and ticks. They would also avoid increased allergy risk, increasing number of heat days and increasing numbers of mosquitos and ticks to a higher degree than allergic tourists would. The latter display a higher avoidance for changing landscape and less certainty of snow on slopes.

## Discussion

This study provides first insights into the perception of and reactions to the ongoing climate change among patients with chronic lung disease and non-patients. While there is growing evidence for the impact of climate change on objective patient outcomes, data on subjective patient estimation is missing so far. Since specific regions and populations are particularly affected by negative consequences of the climate change, we studied patients with chronic lung disease as a vulnerable population in the alpine region of Germany, Italy and Switzerland as a vulnerable location.

When asked about their general perception of being affected by the climate change at present, patients’ and tourists’ mean rating is similar. The similar rating of patients and tourists may have different reasons, however these were not addressed in the study. One reason may be that both groups represent populations that are particularly affected, even though in different ways: Tourists to the alpine regions may be more concerned than the public in general. However, this group was not included in this study.

Interestingly, patients anticipate a less strong increase of impairment in the future than tourists even though projections expect a significantly rising impact [1]. A possible reason could be that they may not have associated this general question with their health.

A main correlating factor with present concern in both groups was allergy. In this regard the high awareness appears in accordance with current clinical investigations and predictions [20,21]. As allergies were of importance, we also compared allergic patients with allergic tourists. Here the pattern of feeling affected in general as well as disturbance and avoidance of specific items, was similar to what was observed in the overall population. Thus, the differences between patients and tourist cannot be explained by different rates of allergy only.

Additionally, in patients the strongest correlation with concern was found for CAT score. As highly symptomatic COPD patients display an increased frequency of exacerbations and a worse prognosis in general [22], their increased concern seems justified. This is also supported by recent data showing correlation between symptoms and climate-change-induced adverse health effects [23].

Importantly, symptoms were significantly ameliorated at the end of rehabilitation as compared to the status before at home emphasizing the benefit of pulmonary rehabilitation in the alpine region in the present patient population.

Of note, in patients we found no correlation with socio-demographic factors, even though older adults are at higher risk especially for heat-wave-related mortality [10,24,25]. Age and education were also significant predictors of risk perception of climate change and heat-waves in a cross-sectional study of the general adult population in Adelaide, Australia [26]. Possible reasons for the lacking age-correlation in the present study, could be the selection of patients with chronic lung disease who are *per se* at higher risk, but also a central-european population that has been less exposed to extreme heat-waves so far. However, this highlights that awareness should be raised especially among older patients.

Regarding specific consequences of climate change, patients were more disturbed by factors that can impair health, such as increased allergy risk, increasing number of mosquitos and decreased air quality which is also corresponding well to the current objective data. Surprisingly, increasing number of heat-days was rated only mildly concerning by patients and also not significantly different from the tourists’ rating despite being known as an important risk factor for increased mortality in chronic respiratory disease [5,6]. Comparing the adaptive behavior of patients and tourists a similar pattern as in the disturbance rating was found. However, here avoidance of heat-days was more pronounced in the patients than in the tourists. Yet, only 53% of the patients would avoid increasing heat-days whereas increasing number of ticks and mosquitos are avoided by a higher percentage (77% and 84% respectively).

Several international and national surveys have addressed the perception of climate change in general and associated health consequences in the general population, but to our knowledge none has focused on patients with lung disease so far. In a national study of American climate change risk perceptions conducted in 2002–3, it was found that mean holistic concern about climate change was 2.9 while current impact’s mean rating was 2.6 on a scale of 1–4 [27]. In our present study using a similar scale of 1–4, mean personal concern at present was lower (2.42 in patients and 2.38 in tourists). Yet, it is difficult to compare these values as the questions asked were not exactly the same. We specifically asked about being affected personally while Leisoriwitz reported about more general concern. At that time, one third of Americans believed climate change to be harmful to people in the US at present and about half thought this would be the case in the future [27], while in our study around 50% of patients and tourists felt already affected at present and much higher rates were found for the future (66% of patients and 83% of tourists). When asked about being affected by climate change in the future the mean rating of the general US population was 2.76 in that study [27] and thus similar to our patient population (2.75) while tourist had higher mean ratings (3.12).

In a survey conducted in the US, Canada and Malta in 2008/9, perception of current and future health risks by climate change was investigated: One third of Americans, one half of Canadians, and two-thirds of Maltese said that people were harmed at present while about half of the Americans thought this would be the case in the future and 15% thought this would never happen [28]. About a third of people in the United States and two thirds of Canadian saw themselves (United States, 32%; Canada, 67%), as being vulnerable to harm from climate change. In this study [28], as well as more recent US national survey [29], relatively few people answered open-ended questions connecting climate change and human health risks. In our study, response rates to specific consequences of climate change including health-related ones were very high in tourist (>99%) and high in patients (>93%). Thus, it can be speculated that respondants had some degree of passive knowledge regarding these items. However, it will be important for our future studies to investigate the level of active knowledge about health risks associated with climate change using open-end questions.

In a survey conducted in German households in 2012 it was found that only 25% of respondants thought that climate change would affect tourism in Germany within the next decades. When asked about consequences of climate change for their personal living conditions, 48.6% of respondants thought that climate change would have very or rather negative consequences, while 48.9% thought that positive and negative consequences were equally important and 2.5% thought that there were rather or very positive consequences. Perception of health risks was not addressed separately in this survey, but together with financial risks. Here it was found that regarding damages of health or finances by heat waves 4% felt affected at present and 52% of participants think damage will occur in Germany in the future. Thus it can be estimated that the degree of feeling affected at present and in the future is higher in our study sample of patients and tourists in the alpine region than in the general German population [30].

### Limitations

This study needs to be interpreted in view of certain limitations. Firstly, as it is a survey that was filled out anonymously, diagnoses of lung disease and allergies are self-reported only and were not verified objectively. This becomes evident as a relatively high number of patients (n = 19, 11%) reported to have both, asthma and COPD. It is unclear if all of these are really affected by Asthma COPD overlap syndrome (ACOS), especially because this diagnosis and its definition is still a matter of debate and research [31–33]. Thus, for further analyses we grouped them together with the COPD group. Also, this COPD group was relatively small (n = 35) rendering statistical analysis less reliable. Moreover, we did not investigate the correlation of data from the survey with objective clinical outcomes.

As expected, the mean age of patients with asthma was younger than the mean age of patients with COPD (50 versus 59 respectively). However, also patients with asthma were mostly middle-aged adults and not young adults as it might be expected from the age prevalence of asthma. This might be due to a selection bias caused by recruitment of the patients in the rehabilitation clinics and therefore the results might not be representative of younger asthma populations. Further bias might be introduced by the time-point of recruitment. While patients were recruited for a longer time-period including the winter, spring and summer season, tourists were recruited in spring and summer season only, thus the main season for pollen allergy.

Secondly, CAT score was used as a measure of symptoms even though the majority of patients who participated had asthma, not COPD. However, there is evidence that the CAT score is also a useful tool in asthma [34–36], while asthma control test (ACT) is disease-specific and focuses on asthma control only.

Thirdly, the control group consisted of tourists who have their own specific intentions for visiting the alpine region and might not be representative of the general population. We estimated this as a valid control group because both, patients undergoing pulmonary rehabilitation and tourists, share the characteristic of being visitors to the alpine region. However, future studies should also include the general population as additional control group to benchmark the patients’ and tourists’ ratings. Moreover baseline socio-demographic characteristics did not differ between patients and tourists except for a small difference in education level, which was higher for tourists. Yet, there were differences in the frequency of allergies, as the majority of patients had allergic asthma.

Finally, the study did not directly ask for the reasons why patients or tourists feel affected by climate change and did not include open-end questions. Even though some conclusions can be drawn by the ratings of the specific consequences of climate change, this needs to be specifically addressed in further detail in future studies.

This study was carried out as a cooperation project between the chair of economic geography and tourism research and the department of respiratory medicine. This report focuses on the analysis of the patient data while tourists without lung disease served as control group. A detailed analysis of the complete tourist dataset will be the subject of another report presented by the chair of economic geography and tourism research.

### Conclusion and future directions

When directly asked about specific health-related consequences of climate change, patients with chronic lung disease are more sensitive to these consequences reflecting their distinct awareness of climate change associated risks, especially concerning allergies. However, the specific hazard of an increasing number of heat-days appears underestimated, and therefore awareness should be raised, particularly in the elderly.

The overall rating of being affected by climate change is similar between patients and tourists at present and even higher in the tourists regarding the future. This point needs to be addressed in future studies to reveal the reasons for these ratings and answer the following research questions: What do patients and tourists associate with climate change in general? What is their knowledge about health-related consequences of climate change? Why do they feel affected? What are their main concerns regarding different specified time-points in the future? How do these ratings of patients and tourists compare to the general public?

## Supporting information

S1 Fig General perception of being affected by climate change in patients and tourists with allergies. Both groups anticipated to be significantly more affected in the future than at present (Fisher’s Exact test, Patients: p<0.05; Tourists: p<0.001). In the future allergic tourists feel more affected than allergic patients (Fisher’s Exact test, patients p<0.05, tourists p<0.001).
(TIFF)

S2 Fig Disturbance and avoidance of specific consequences of climate change in patients and tourists with allergies. Left: Degree of disturbance. Rating: 1- not disturbing, 2- rather disturbing, 3- disturbing, 4 absolutely disturbing. Right: Avoidance. Rating: 1- not avoid, 2- avoid. The item extreme weather events include thunderstorms, gale storms and heavy rain, but not heat days. * p<0.05, ** p<0.01, *** p<0.001 significant differences between patients and tourists by Kruskal-Wallis-Test.
(TIFF)

S1 File Survey English. (PDF)

S2 File Survey German. (PDF)

S3 File Source Data. (ZIP)
