# Supplementary figures and images for: Perception of climate change in patients with chronic lung disease
Source: PLoS One. 2017 Oct 18;12(10):e0186632. doi: 10.1371/journal.pone.0186632 (PMC5646841; doi:10.1371/journal.pone.0186632)

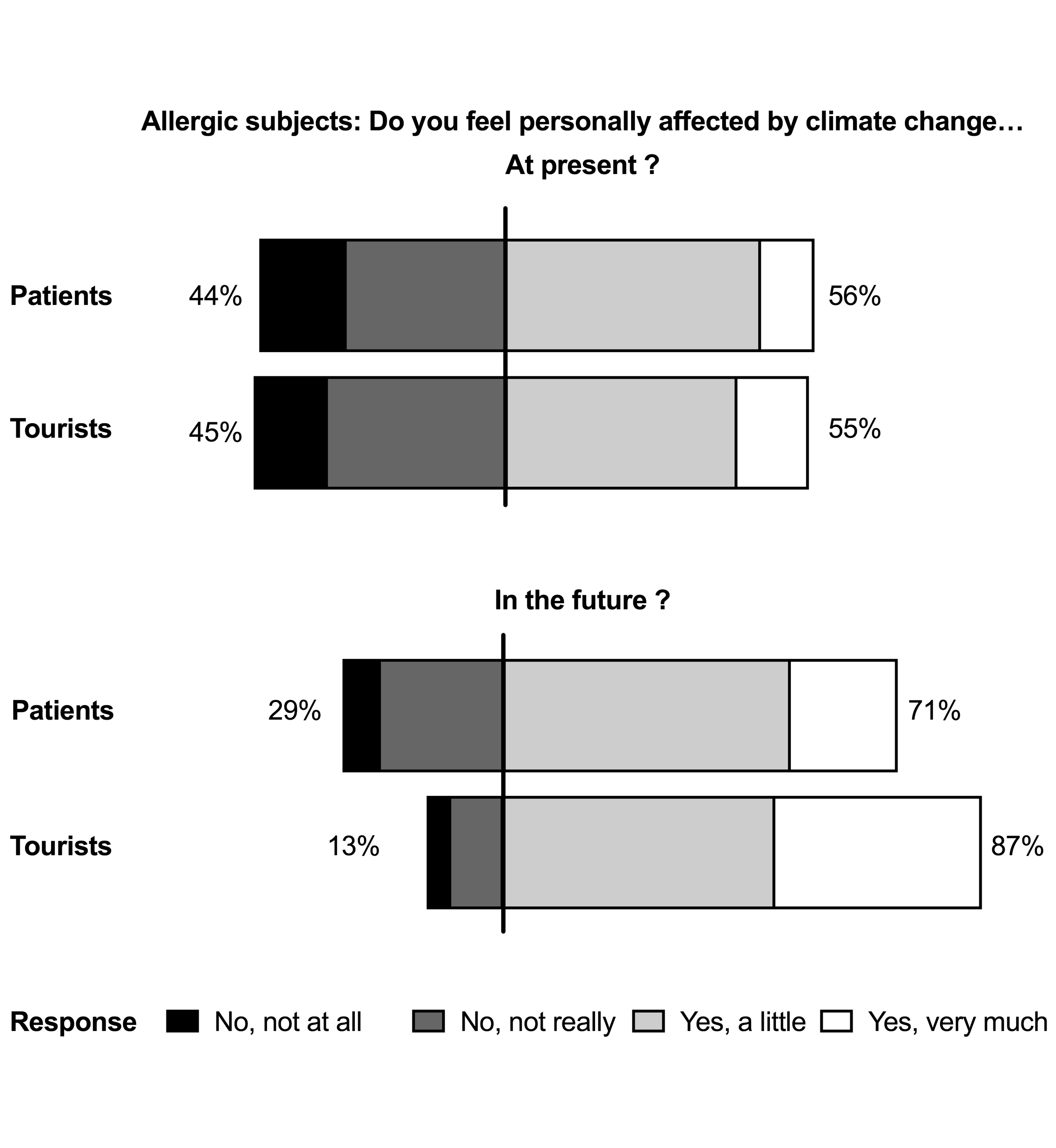

Supplement: S1 Fig — Both groups anticipated to be significantly more affected in the future than at present (Fisher’s Exact test, Patients: p<0.05; Tourists: p<0.001). In the future allergic tourists feel more affected than allergic patients (Fisher’s Exact test, patients p<0.05, tourists p<0.001). (TIFF) [file pone.0186632.s001.tiff]

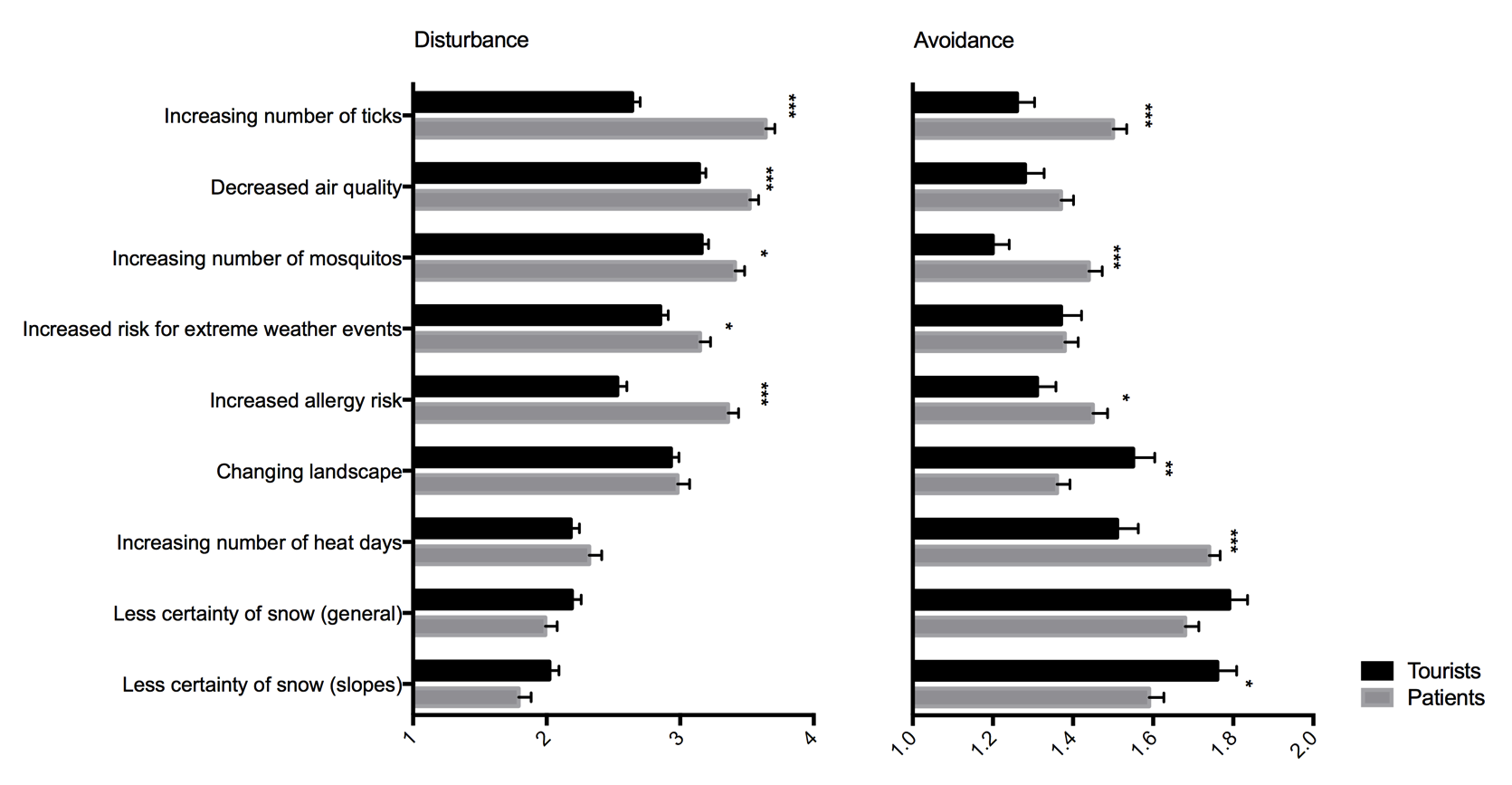

Supplement: S2 Fig — Left: Degree of disturbance. Rating: 1- not disturbing, 2- rather disturbing, 3- disturbing, 4 absolutely disturbing. Right: Avoidance. Rating: 1- not avoid, 2- avoid. The item extreme weather events include thunderstorms, gale storms and heavy rain, but not heat days. * p<0.05, ** p<0.01, *** p<0.001 significant differences between patients and tourists by Kruskal-Wallis-Test. (TIFF) [file pone.0186632.s002.tiff]
