# Supplementary material for: Perception of climate change in patients with chronic lung disease
Source: PLoS One. 2017 Oct 18;12(10):e0186632. doi: 10.1371/journal.pone.0186632 (PMC5646841; doi:10.1371/journal.pone.0186632)
Supplement: S1 File — (PDF) [file pone.0186632.s003.pdf]

Survey of climate change and health in alpine regions		
**1**	1. **Do you currently smoke cigarettes regularly (even if only few)?**<br><br>* by regularly we mean:<br>1 cigarette per day,<br>or at least 5 cigarettes per week,<br>or at least 1 pack of cigarettes per month for at least 6 months	*Please mark with a cross (x) or fill in (___) as appropriate.*<br><br>❑ Yes<br><br>➢ How much do you smoke at the moment? Number of cigarettes per day: ________<br><br>➢ When did you start to smoke regularly? At what age or in which year ________<br><br>❑ No, I no longer smoke, but I used to be a regular smoker<br><br>➢ How much did you used to smoke? Number of cigarettes per day: _______<br><br>➢ When did you become a regular smoker? At what age or in which year? ________<br><br>➢ When did you stop smoking? At what age or in which year ________<br><br>❑ No, I have never smoked regularly *.
	**Have you ever been diagnosed with a lung disease by a doctor?**	*Please mark with a cross (x) or fill in (___) as appropriate*<br><br>❑ Yes<br><br>➢ Which lung disease?<br><br>❍ Asthma<br><br>❍ Chronic bronchitis/ Chronic obstructive pulmonary disease (COPD)/Emphysema<br><br>❍ Lung fibrosis<br><br>❍ I don't know the exact name of the illness<br><br>❍ Other: __________________________<br><br>❑ No
	**Do you suffer from hay fever?**	*Please mark with a cross (x).*<br><br>❑ Yes ❑ No
**4**	**Have you been diagnosed with an allergy?**	*Please mark with a cross (x) or fill in (___) as appropriate*

		❑ Yes<br>➢ Which? ______________________<br>❑ No

**5**	**Do you take tablets regularly?**	*Please mark with a cross (x).*<br>❑ Yes<br>➢ Do you use inhalers regularly? ❍Yes ❍No<br>❑ No

Symptoms suffered at home in the previous 3 months: For each of the following statements, please pick the option that best fits in your daily life at home. For each statement, please place only one mark.

**6**<br>……	Level	↔						Level
	I never cough	0	1	2	3	4	5	I cough all the time.
	I have no phlegm (mucus) in my chest at all	0	1	2	3	4	5	My chest is full of phlegm (mucus)
	My chest does not feel tight at all	0	1	2	3	4	5	My chest feels very tight
	When I walk up a hill or one flight of stairs I am not breathless	0	1	2	3	4	5	When I walk up a hill or one flight of stairs I am very breathless
	I am confident leaving my home despite my lung condition	0	1	2	3	4	5	I am not at all confident leaving my home because of my lung condition
	I sleep soundly	0	1	2	3	4	5	I don't sleep soundly because of my lung condition
	I have lots of energy	0	1	2	3	4	5	I have no energy at all

Symptoms during your current stay: For each statement, please pick the option that best describes you currently. For each statement, please place only one mark.

7<br>……	Level	↔						Level
	I never cough	0	1	2	3	4	5	I cough all the time.
	I have no phlegm (mucus) in my chest at all	0	1	2	3	4	5	My chest is full of phlegm (mucus)

	My chest does not feel tight at all	0	1	2	3	4	5	My chest feels very tight
	When I walk up a hill or one flight of stairs I am not breathless	0	1	2	3	4	5	When I walk up a hill or one flight of stairs I am very breathless
	I am confident leaving my home despite my lung condition	0	1	2	3	4	5	I am not at all confident leaving my home because of my lung condition
	I sleep soundly	0	1	2	3	4	5	I don't sleep soundly because of my lung condition
	I have lots of energy	0	1	2	3	4	5	I have no energy at all

**8**	**Do you feel personally affected by climate change at the moment?**	*Please mark the most appropriate answer (❑).*<br>❑ No, not at all ❑ No, not really<br>❑ Yes, a littel ❑ Yes, very much
**9**	**Do you believe that you will be personally affected by climate change in the future?**	*Please mark the most appropriate answer (❑).*<br>❑ No, not at all ❑ No, not really<br>❑ Yes, a littel ❑ Yes, very much

**10**	**When you consider your current stay, how disturbing would the following changes in the alps be for you personally?**<br><br>**And if disturbing: Would you avoid places with such changes in the future?**	*Please mark the most appropriate answer (❑) or fill in (__)*					
		Disturbing				Avoid	
		**Not disturbing**	**Rather disturbing**	**Disturbing**	**Absolutely disturbing**	**I would avoid**	**I would not avoid**
	An increase in the number of hot days (of at least 30° C)	❑	❑	❑	❑	❑	❑
	Less snow on the ski slopes	❑	❑	❑	❑	❑	❑
	Less snow overall	❑	❑	❑	❑	❑	❑
	Increasing number of mosquitos	❑	❑	❑	❑	❑	❑
	Increasing number of ticks	❑	❑	❑	❑	❑	❑
	Increased risk of allergy (e.g., pollen or grass)	❑	❑	❑	❑	❑	❑

	Decrease in air quality	❑	❑	❑	❑	❑	❑
	Change in the landscape (e.g. monocultures, woods instead of pastures etc.)	❑	❑	❑	❑	❑	❑
	Increased risk of extreme weather conditions (e.g.rain, gale storms and thunderstorms etc.)	❑	❑	❑	❑	❑	❑
	Other: ___________________________	❑	❑	❑	❑	❑	❑

General personal information (all the information collected in this questionnaire will remain strictly confidential and will be processed anonymously).

11	**What is your highest educational qualification?**	*Please mark the most appropriate answer (❑) or fill in (__)*<br>❑ no qualification<br>❑ primary school<br>❑ Secondary school (GCSE level),<br>❑ advanced technical college entrance qualification<br>❑ diploma from German secondary school qualifying for university admission<br>❑ college diploma<br>❑ university diploma,<br>❑ other (e.g. doctoral degree): ___________________________
12	**To which sex do you belong?**	Please mark with a cross as appropriate (q).<br>❑ Female ❑ Male
13	**How old are you?**	*Please fill in (___).*<br>Age in years: _______

Thank you for your help!
