# Supplementary material for: Perception of climate change in patients with chronic lung disease
Source: PLoS One. 2017 Oct 18;12(10):e0186632. doi: 10.1371/journal.pone.0186632 (PMC5646841; doi:10.1371/journal.pone.0186632)
Supplement: S2 File — (PDF) [file pone.0186632.s004.pdf]

Umfrage zum Thema Klimawandel und Gesundheit im Alpenraum		
**1**	**Rauchen Sie zurzeit regelmäßig Zigaretten – wenn auch nur wenige?**<br><br>* Unter regelmäßig verstehen wir:<br><br>Mind. 1 Zigarette pro Tag oder mindestens 5 Zigaretten pro Woche oder mindestens 1 Packung Zigaretten pro Monat für mindestens 6 Monate	*Zutreffendes bitte ankreuzen (❑) bzw. eintragen (_).*<br><br>❑ Ja<br><br>➢ ·Wie viel rauchen Sie derzeit gewöhnlich? Anzahl Zigaretten pro Tag: ________<br><br>➢ ·Wann haben Sie angefangen, regelmäßig* zu rauchen? Im Alter von Jahren bzw. im Jahr ________<br><br>❑ Nein, ich habe früher regelmäßig* geraucht, aber jetzt nicht mehr<br><br>➢ ·Wie viel haben Sie früher gewöhnlich geraucht? Anzahl Zigaretten pro Tag:______<br><br>➢ ·Wann haben Sie angefangen, regelmäßig* zu rauchen? Im Alter von Jahren bzw. im Jahr ________<br><br>➢ ·Wann haben Sie aufgehört zu rauchen? Im Alter von Jahren bzw. im Jahr ________<br><br>❑ Nein, ich habe noch nie regelmäßig* geraucht
**2**	**Wurde bei Ihnen jemals von einem Arzt eine chronische Lungenerkrankung diagnostiziert?**	*Zutreffendes bitte ankreuzen (❑und/oder❍) bzw. eintragen (___).*<br><br>❑ Ja<br><br>➢ Wie heißt diese Lungenerkrankung?<br>❍ Asthma<br><br>❍ Chronische Bronchitis/Chron. obstruktive Lungenerkrankung (COPD)/Emphysem<br><br>❍ Lungenfibrose<br><br>❍ genaue Bezeichnung ist mir nicht bekannt<br><br>❍ Sonstige: __________________________<br><br>❑ Nein
**3**	**Leiden Sie unter Heuschnupfen?**	*Zutreffendes bitte ankreuzen (❑).* ❑ Ja ❑ Nein
**4**	**Wurde bei Ihnen eine Allergie festgestellt?**	*Zutreffendes bitte ankreuzen (❑) bzw. eintragen (_).*<br>❑ Ja<br><br>➢ Welche? ________________________________<br><br>❑ Nein

**5**	**Nehmen Sie regelmäßig Medikamente ein?**	*Zutreffendes bitte ankreuzen (❑und/oder❍).*<br>❑ Ja<br>➢ Nehmen Sie regelmäßig inhalative Medikamente ein? ❍ Ja ❍ Nein<br>❑ Nein

Symptome in den letzten 3 Monaten zu Hause: Bitte geben Sie für jede der folgenden Aussagen an, was für Sie zuletzt zu Hause am besten zutrifft. Kreuzen Sie in jeder Zeile bitte nur eine Möglichkeit an.

**6** …….	*Ausprägung*	⟷						*Ausprägung*
	Ich huste nie	0	1	2	3	4	5	Ich huste immer
	Ich bin überhaupt nicht verschleimt	0	1	2	3	4	5	Ich bin völlig verschleimt
	Ich spüre keinerlei Engegefühl in der Brust	0	1	2	3	4	5	Ich spüre starkes Engegefühl in der Brust
	Wenn ich bergauf oder eine Treppe hinaufgehe, komme ich nicht leicht außer Atem	0	1	2	3	4	5	Wenn ich bergauf oder eine Treppe hinaufgehe, komme ich leicht außer Atem
	Ich habe keine Bedenken trotz meiner Luftnot, das Haus zu verlassen	0	1	2	3	4	5	Ich habe wegen meiner Luftnot große Bedenken, das Haus zu verlassen
	Ich schlafe tief und fest	0	1	2	3	4	5	Ich schlafe nicht tief und fest
	Ich bin voller Energie	0	1	2	3	4	5	Ich habe überhaupt keine Energie

Symptome während des aktuellen Urlaubs/Aufenthalts hier: Bitte geben Sie für jede der folg. Aussagen an, was für Sie aktuell am besten zutrifft. Kreuzen Sie in jeder Zeile bitte nur eine Möglichkeit an.

**7** …….	*Ausprägung*	⟷						*Ausprägung*
	Ich huste nie	0	1	2	3	4	5	Ich huste immer
	Ich bin überhaupt nicht verschleimt	0	1	2	3	4	5	Ich bin völlig verschleimt
	Ich spüre keinerlei Engegefühl in der Brust	0	1	2	3	4	5	Ich spüre starkes Engegefühl in der Brust
	Wenn ich bergauf oder eine Treppe hinaufgehe,	0	1	2	3	4	5	Wenn ich bergauf oder eine Treppe hinaufgehe,

	komme ich nicht leicht außer Atem							komme ich leicht außer Atem
	Ich habe keine Bedenken trotz meiner Luftnot das Haus zu verlassen	0	1	2	3	4	5	Ich habe wegen meiner Luftnot große Bedenken das Haus zu verlassen
	Ich schlafe tief und fest	0	1	2	3	4	5	Ich schlafe nicht tief und fest
	Ich bin voller Energie	0	1	2	3	4	5	Ich habe überhaupt keine Energie

**8**	**Fühlen Sie sich momentan persönlich durch den Klimawandel betroffen?**	*Zutreffendes bitte ankreuzen (❑).*<br>❑ Nein, überhaupt nicht ❑ Nein, eher nicht<br>❑ Ja, eher ❑ Ja, sehr					
**9**	**Glauben Sie, dass Sie zukünftig persönlich durch den Klimawandel betroffen sein werden?**	*Zutreffendes bitte ankreuzen (❑).*<br>❑ Nein, überhaupt nicht ❑ Nein, eher nicht<br>❑ Ja, eher ❑ Ja, sehr					
**10**<br>.....<br>.....<br>.	**Wenn Sie an Ihren jetzigen Aufenthalt hier denken, wie störend sind bzw. wären für Sie persönlich folgende Veränderungen in den Alpen?**<br><br>**Und falls störend: Würden Sie Orte mit derartigen Veränderungen meiden?**	*Zutreffendes bitte ankreuzen (❑) bzw. eintragen (_).*					
		Stören				Meiden	
		**Nicht störend**	**Eher störend**	**Stören d**	**Absolut störend**	**Würde ich meiden**	**Würde ich nicht meiden**
	Zunahme von Hitzetagen (d.h. mind. 30° C)	❑	❑	❑	❑	❑	❑
	Weniger Schnee auf Skipisten	❑	❑	❑	❑	❑	❑
	Weniger Schnee allgemein	❑	❑	❑	❑	❑	❑
	Verstärktes Auftreten von Stechmücken	❑	❑	❑	❑	❑	❑
	Verstärktes Auftreten von Zecken	❑	❑	❑	❑	❑	❑
	Erhöhtes Allergierisiko (z.B. Pollen oder Gräser)	❑	❑	❑	❑	❑	❑
	Abnahme der Luftqualität	❑	❑	❑	❑	❑	❑
	Veränderung des Landschaftsbildes (Monokulturen, Wälder statt Almen etc.)	❑	❑	❑	❑	❑	❑

	Erhöhtes Risiko für Extremwetterereignisse (Regen, Stürme, Unwetter etc.)	❑	❑	❑	❑	❑	❑
	Sonstiges: ____________________________	❑	❑	❑	❑	❑	❑

**Allgemeine Angaben zur Person** (alle Angaben im Fragebogen werden streng vertraulich behandelt und anonymisiert verarbeitet)		
**11**	**Was ist Ihr höchster Bildungsabschluss?**	*Zutreffendes bitte ankreuzen (❑) bzw. eintragen (__).*<br>❑ Kein Abschluss<br>❑ Haupt-/Volksschule<br>❑ Mittlere Reife/Realschule<br>❑ Fachhochschulreife<br>❑ Abitur<br>❑ Fachhochschule<br>❑ Hochschule<br>❑ Sonstiges: ________________________________
**12**	**Welchem Geschlecht gehören Sie an?**	*Zutreffendes bitte ankreuzen (❑).*<br>❑ Weiblich ❑ Männlich
**13**	**Und zu guter Letzt, wie alt sind Sie?**	*Zutreffendes bitte eintragen (___).*<br>Alter in Jahren: _______

Vielen Dank für Ihre Unterstützung!
